# Bacterial diversity and reductive dehalogenase redundancy in a 1,2-dichloroethane-degrading bacterial consortium enriched from a contaminated aquifer

**DOI:** 10.1186/1475-2859-9-12

**Published:** 2010-02-19

**Authors:** Massimo Marzorati, Annalisa Balloi, Francesca de Ferra, Lorenzo Corallo, Giovanna Carpani, Lieven Wittebolle, Willy Verstraete, Daniele Daffonchio

**Affiliations:** 1 DISTAM, Dipartimento di Scienze e Tecnologie alimentari e Microbiologiche, Università degli Studi di Milano, 20133, Milan, Italy; 2 Eni DIV, R&M, 20097, San Donato Milanese, Italy; 3 Laboratory for Microbial Ecology and Technology (LabMET), Ghent University, B9000 Ghent, Belgium

## Abstract

**Background:**

Bacteria possess a reservoir of metabolic functionalities ready to be exploited for multiple purposes. The use of microorganisms to clean up xenobiotics from polluted ecosystems (e.g. soil and water) represents an eco-sustainable and powerful alternative to traditional remediation processes. Recent developments in molecular-biology-based techniques have led to rapid and accurate strategies for monitoring and identification of bacteria and catabolic genes involved in the degradation of xenobiotics, key processes to follow up the activities *in situ*.

**Results:**

We report the characterization of the response of an enriched bacterial community of a 1,2-dichloroethane (1,2-DCA) contaminated aquifer to the spiking with 5 mM lactate as electron donor in microcosm studies. After 15 days of incubation, the microbial community structure was analyzed. The bacterial 16S rRNA gene clone library showed that the most represented phylogenetic group within the consortium was affiliated with the phylum *Firmicutes*. Among them, known degraders of chlorinated compounds were identified. A reductive dehalogenase genes clone library showed that the community held four phylogenetically-distinct catalytic enzymes, all conserving signature residues previously shown to be linked to 1,2-DCA dehalogenation.

**Conclusions:**

The overall data indicate that the enriched bacterial consortium shares the metabolic functionality between different members of the microbial community and is characterized by a high functional redundancy. These are fundamental features for the maintenance of the community's functionality, especially under stress conditions and suggest the feasibility of a bioremediation treatment with a potential prompt dehalogenation and a process stability over time.

## Background

Ecological models and experimental data suggest that taxonomic diversity and genetic-functional redundancy are key factors in increasing the flexibility of microbial communities that subsequently can both perform a given metabolic function more efficiently and better overcome metabolic stresses [1-3]. Biodiversity is linked to both the chemical-physical characteristics of the environment and the dynamic and synergistic interactions between microbes that in turn contribute to determine the level of ecosystem functioning [4-6]. The ecological behavior of microbes together with their enormous metabolic potential is a valuable solution to remediation of polluted environments [7-12]. The exploitation of the enormous metabolic capabilities associated with microorganisms has been recently defined as Microbial Resource Management (MRM): bacteria can be seen as cell factories that are stirred to produce or degrade a given compound under human management [13].

Among major anthropogenic pollutants, chlorinated aliphatics and aromatics are of great concern especially in anaerobic environments like groundwater and sediments where they tend to accumulate. For example, 1,2-dichloroethane (1,2-DCA) is considered a priority pollutant, being the most abundant double-carbon chlorinated compound contaminating the groundwater worldwide [14,8]. Literature reports several examples on how microbial metabolic potential can enhance the removal of 1,2-DCA [10-12] as well as of other chlorinated alkanes and alkenes [8,15-17]. Key enzymes implicated in the dechlorination processes are the reductive dehalogenases (RDs), a class of enzymes capable of eliminating chlorine from molecules [18-24].

In our previous study, RD sequences correlated with 1,2-DCA dechlorination to ethene have been identified in *Desulfitobacterium dichloroeliminans *strain DCA1 (RD DCA1) - a microorganism that can efficiently dechlorinate 1,2-DCA using hydrogen as electron donor - and *in situ *in a 1,2-DCA contaminated aquifer (RD 54) [12,25]. The enrichment culture setup from the aquifer (culture 6VS), was reported to contain both *Dehalobacter *and *Desulfitobacterium *spp. Recently three novel RDs sequences (WL *rdhA1*, WL *rdhA2 *and WL *rdhA3*) have been identified in a 1,2-DCA dehalogenating enrichment culture containing *Dehalobacter *sp. WL [26]. In the present work, the 6VS mixed culture [25] was structurally and functionally characterized after transferring in fresh anaerobic microcosms amended with 1,2-DCA and lactate as electron donor. The final aim was to describe the response of the resident microbial community, in terms of species diversity and functional redundancy, in order to foresee a biostimulation or a bioaugmentation treatment to solve the problem *in situ*.

## Results

### Bacterial diversity

We recently described a groundwater aquifer with a historical contamination by 1,2-DCA that held dechlorinating microorganisms [12,25]. The bacterial dehalogenating consortium was maintained through adhesion of the original culture to an active-carbon support [25] and used to set up a new microcosm. The microbial community was incubated for 15 days, in anaerobic conditions, in presence of 5 mM of lactate as electron donor and 900 mg L^-1 ^of 1,2-DCA. Following lactate amendment, the 1,2-DCA was completely dechlorinated in about 260 h (11 days). To evaluate the presence in the microbial community of species potentially involved in the 1,2-DCA degradation, we examined the diversity of microbial communities by establishing 16S rRNA gene clone libraries. PCR with specific primers for archaea did not give any amplicon, even after a second round of PCR using nested primers.

**Figure 1:**
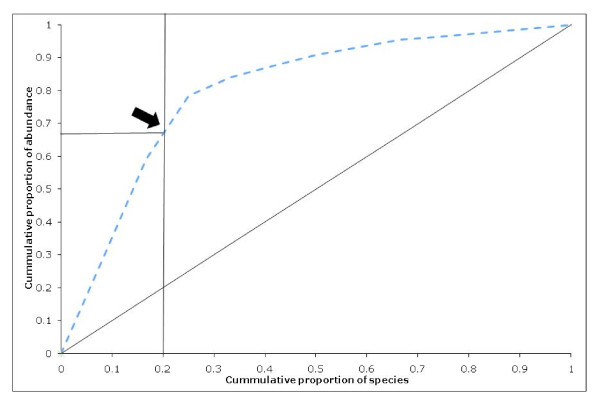
**Pareto-Lorenz distribution of bacterial diversity**. Pareto-Lorenz distribution curve of the microbial community's evenness after the biostimulation treatment. The 45° diagonal represents a perfect evenness of a community. The black arrow indicates the OTU cumulative proportion of abundances for an OTU cumulative proportion of 0.2.

The overall community diversity as a function of both the total number of species present (richness) and their relative distribution (evenness) was calculated from the data obtained from the bacterial 16S rRNA gene libraries after lactate amendment. The diversity and structure of bacterial community was analyzed using three indices: 1) Species richness, that is the number of species identified in a sample; 2) Simpson's index, that is the probability that two clones, taken at random from a sample, will be the same species. This measure is sensitive to changes in the frequency of the more abundant species; 3) Shannon-Wiener index, that reflects the diversity and the evenness of clones distribution between taxa. It is sensitive to changes in the frequency of common and less common (though not the rarer) species [27]. After the treatment, the bacterial community of the enrichment culture was characterized by a relatively high species richness (12 species), with a Simpson index of 0.781 and a Shannon index of 1.807. Pareto-Lorenz curve was used as a graphical estimator of the species evenness [28,29]. The operational taxonomic units (OTUs) were ranked from high to low, based on their relative abundance within the library. The cumulative normalized number of clones was used as X-axis, and their respective cumulative proportion of abundances was reported in the Y-axis (Figure 1A). Following the treatment, Pareto-Lorenz curves showed a situation where 20% of the OTUs represented 65-70% of the total abundance of clones.

A summary of the clones identified in the libraries is shown in Table 1. The 12 OTUs are reported with the percent distribution normalized by rRNA gene copy numbers from the closest phylogenetic relatives, in order to minimize the rRNA copy number bias [30]. Rarefaction curves to saturation indicated that microbial diversity in the library was satisfactorily covered (data not shown). The bacterial clone libraries were dominated by sequences related to *Desulfitobacterium*, *Dehalobacter *and *Clostridium *genera, in the *Firmicutes *phylum. In particular the most abundant sequences were those identified having a 97% similarity with *Desulfitobacterium dichloroeliminans *strain DCA1 and a 99% similarity with *Dehalobacter *sp. WL (26 clones for each sequence over a total of 88 clones). Both these bacteria have been reported to dechlorinate 1,2-DCA to ethene [25,26]. Hence, the microbial community resulted to be dominated by few species (Figure 1A) and most of them are potentially related to the degradation of chlorinated compounds (Figure 1B). Other numerically relevant clones were related to *Desulfitobacterium metallireducens *and to *Trichlorobacter thiogenes*.

**Table 1:** 16S rRNA gene OTUs in the groundwater bacterial community 15 days after lactate amendment

OTU^a^	N. of clones	Accession Number of identified OTUs	Closest database hit	Nt identity and Reference	Phylum	Copies 16S rRNA gene^b^	Normalized % over total clones^c^	Characteristics of the closest described relative	Source
1**	26	*FM205003*	*Desulfitobacterium dichloroeliminans *[AJ565938]	956/978 (97%)	Firmicutes	6	26.6 - 38.2	1,2 DCA dechlorinating bacterium	[37]
2**	26	FM204994	*Dehalobacter *sp. WL [DQ250129]	1480/1489 (99%)	Firmicutes	5 - 12	19.1 - 32.0	Chlorinated ethanes and 1,2 DCA dechlorinating bacterium	[35]
3**	17	*FM205004*	*D. metallireducens *[AF297871]	1408/1459 (96%)	Firmicutes	6	17.4 - 25.0	Couples growth to the reduction of metals and humic acids as well as chlorinated compounds	[36]
4	5	FM205000	Uncult Clostridiaceae [AF255644]	1393/1448 (96%)	Firmicutes	5 - 12	3.7 - 6.1	Fermentative obligate anaerobe isolated from black mud	[49]
5*	2	FM204997	*Trichlorobacter thiogenes *[AF223382]	1466/1484 (98%)	δ-Proteobacteria	4	3.1 - 4.4	Reductive dehalogenating bacteria	[50]
6*	3	FM204996	Uncult. Clostridium [AB186865]	1413/1426 (99%)	Firmicutes	5 - 12	2.2 - 3.7	Acetogenic bacterium from acidic sediments	Drake et al., (Unpublished)
7*	3	*FM205001*	*Clostridium *sp. CYP5 [DQ479415]	1430/1432 (99%)	Firmicutes	5 - 12	2.2 - 3.7	sulfur-reducing anaerobe isolated from an olive mill wastewater contaminated by phosphogypse	Ben Dhia et al., (Unpublished)
8	2	FM204995	Uncult Clostridiaceae [AF255644]	1347/1444 (93%)	Firmicutes	5 - 12	1.5 - 2.5	Fermentative obligate anaerobe isolated from black mud	[49]
9	1	FM204999	Uncult. Bacteroidetes [AJ488070]	1446/1450 (99%)	Bacteroidetes	5 - 6	1.2 - 1.5	Propionate-producing bacterium isolated from plant residue in irrigated rice-field	[51]
10	1	*FM204998*	*Clostridium saccharolyticum *[Y18185]	1414/1460 (96%)	Firmicutes	5 - 12	0.7 - 1.2	Bacteria able to convert a number of mono and disaccharides to ethanol, acetic acid, lactic acid, hydrogen and carbon dioxide	[52]
11	1	FM205002	Uncult clone PL-5B10 [AY570634]	1404/1460 (96%)	Firmicutes	5 - 12	0.7 - 1.2	Thiosulfate-reducing bacterium from an oil-producing well	[53]
12*	1	FM205005	Uncult Clostridiaceae [AJ009473]	1359/1447 (93%)	Bacteroidetes	5 - 12	0.7 - 1.2	Obligate anaerobic mesophile bacterium isolated from a UASB reactor	Song and Dong (Unpublished)

^a ^* indicates those clones related to microorganisms identified in community degrading chlorinated compounds; ** indicate those clones related to microorganisms that are well known degraders of chlorinated compounds.

^b ^Based on numbers reported for the same genus or close relatives [54,55,30];

^c ^Percentage of clones normalized for estimated rRNA gene copy number according to numbers reported for the same genus or close relatives

### Functional genes diversity

**Figure 2:**
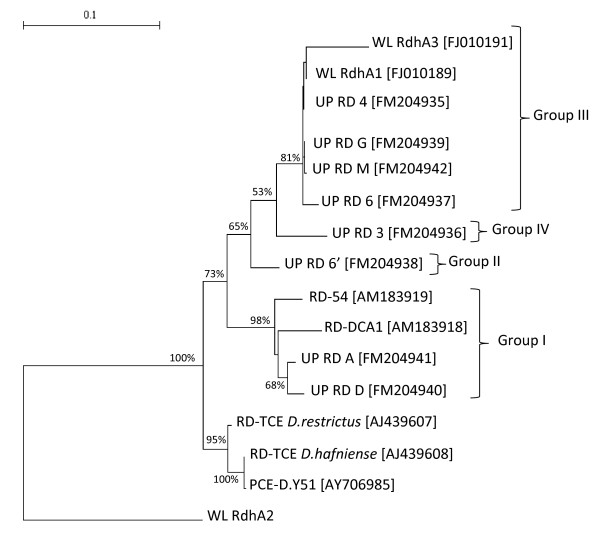
**RDs sequences phylogenetic tree**. Neighbour-joining tree with branch lengths to assess the relationship between DcaA of a previously characterized RD from the contaminated aquifer (RD-54) [Accession number: AM183919] and of *D. dichloroeliminans *strain DCA1 (RD-DCA1) [AM183918] with other A subunits of already characterized RDs, PceA of *Dehalobacter restrictus *strain DSMZ 9455^T ^(RD-TCE *D. restrictus*) [AJ439607], *Desulfitobacterium hafniense *strain TCE1 (RD-TCE *D. hafniense*) [AJ439608] and *Desulfitobacterium *sp. strain Y51 (PCE-D.Y51) [AY706985]. Besides, are also reported the recently identified WL RdhA1, WL RdhA2 and WL RdhA3 [FJ010189, FJ010190, FJ010191] and the RDs identified in enrichment culture (UP RD X). The numbers at each node represent percentage of bootstrap calculated from 1000 replicate trees. The scale bar represents the sequence divergence.

In order to evaluate the functional redundancy of the resident microbial community in response to biostimulation with lactate, the reductive dehalogenase diversity was investigated. For this purpose, functional gene libraries of this class of enzymes were prepared starting from the total DNA extracted from the microcosm. Primers PceAFor1 and DcaB rev, annealing upstream and downstream the gene *dcaA*, were used to amplify the variable region of the *dcaABCT *gene cluster coding for the reductive dehalogenase [25]. A total of 39 clones were sequenced and sequences were aligned with those from previously characterized RD. The phylogenetic relationship among the different RDs found in the groundwater is shown in Figure 2. Four groups (I-IV) of RDs could be identified. Most of the clones were affiliated to groups I and III, while groups II and IV were represented by only two clones each. A high proportion of clones (24 over a total of 39) clustered in group I and were closely related to the RD previously identified in *D. dichloroeliminans *strain DCA1 (RD DCA1) and in the same contaminated site (RD 54) (the nucleotide identity was higher than 98%). The sequence UP-RD-6', representing the RD group II, was related to the RD of the strain DCA1 but with a lower nucleotide identity (96%). A third group, including the sequences of eleven clones, presented only a 94% and a 88-89% identity at respectively nucleotide and amino acid level with the DcaA catalytic subunit previously identified in the same aquifer [25]. On the contrary, these sequences had an amino acid identity between 98 and 99% with WL *rdhA1*, one of the three RDs sequences recently identified by Grostern and colleagues [26] in *Dehalobacter *sp. WL.

**Figure 3:**
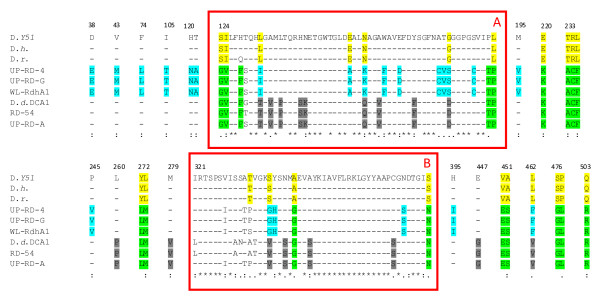
**Amino acid alignment of the DcaA proteins**. Amino acid alignment of the DcaA proteins of groups I (UP-RD-A) and III (UP-RD-4 and UP-RD-G) of the new identified RDs, with: those previously identified in the groundwater (RD-54), in *D. dichloroeliminans *strain DCA1 (D.d. DCA1) [Accession number AM183919 and AM183918], with PceA of *Desulfitobacterium *sp. strain Y51 (D. Y51) [AY706985], *D. hafniense *strain TCE1 (D.h.) [AJ439608], *D. restrictus *strain DSMZ 9455T (D.r.) [AJ439607], and with the recently identified WL *rdhA1 *(WL-RdhA1) [FJ010189]. Only the regions including amino acid variations among the different proteins are presented. Red rectangles A and B indicate the protein stretches where it has been previously indicated that resides 53% of the total amino acid diversity between DcaA and PceA [25]. Light blue residues are amino acids specific for the DcaA of the group III, proposed to be specific for the 1,2-DCA RD from *Dehalobacter*; dark grey residues those specific for the DcaA subunit of the group I, proposed to be specific for the 1,2-DCA RD from *Desulfitobacterium*; green residues those common to all the RDs identified in the aquifers contaminated by 1,2-DCA but not conserved in the PCE-specific RDs. Asterisks, colons and dots below the alignment indicate an identical position in all the proteins, a position with a conservative substitution and a position with a semi-conservative substitution, respectively.

It has been previously shown that 53% of the total amino acid diversity of DcaA of the RD-54 and RD-DCA1, when aligned with PceA of RDs specific for tetrachloroethene (PCE) from *Dehalobacter restrictus *strain DSMZ 9455^T ^[31], *Desulfitobacterium sp*. strain Y51 [21], and *Desulfitobacterium hafniense *strain PCE-S [20], was mainly localized in two small regions (blocks A and B - Figure 3) that represent only 19% (104 amino acids over 551) of the total DcaA residues [25]. These two regions of hyper-variability were proposed to be involved in 1,2-DCA recognition or in general in the substrate specificity of RDs [25]. The novel RDs sequences, identified in this work, were aligned to WL *rdhA1 *and to sequences of the previously mentioned RDs specific for PCE. From the alignment it was possible to identify two hyper-variable regions overlapping with blocks A and B (Figure 3). In particular we focused our attention on the RDs sequences of groups I and III, considering the higher number of clones in these two clusters. Within blocks A and B of the selected RDs sequences, as well in other smaller regions of the DcaA subunit, it was possible to identify amino acids specific for: i) PceA, the RDs specific for PCE (yellow residues); ii) DcaA of group III, an RD from *Dehalobacter *proposed to be specific for 1,2-DCA (light blue residues), iii) DcaA of the group I, an RD from *Desulfitobacterium*, proposed to be specific for 1,2-DCA (dark grey residues), iv) all the RDs within the groups I and III but not for PCE-specific RDs (green residues).

## Discussion

The stability of microbial communities has been suggested to correlate not only to the number of species occupying a niche but also to the functional redundancy found in the same niche [32,33]. Under this concept, microbial communities should be more flexible if the same function prevails under varying environmental conditions and is supported by enzymes adapted to the different conditions the microbial community is exposed to. In this study, a reductive dechlorination activity was linked to an adapted and specialized bacterial community that presented, after suitable stimulation, a high functional redundancy. According to the three diversity indexes (species richness, Simpson and Shannon index) and the Pareto Lorenz curve representation, the bacterial community is characterized by a good richness and few dominant species. Following the interpretation reported by Marzorati and colleagues [29], the structure of the studied microbial community (the 20% of the OTUs represent the 65-70% of the total abundance of clones) can be considered typical of a specialized community (as an enriched microbial community should be). The 16S rRNA gene clone library showed that the 1,2-DCA-degrading consortium was equally dominated by two different taxa related to *Desulfitobacterium dichloroeliminans *strain DCA1 and *Dehalobacter *sp. WL., previously shown to be involved in the 1,2-DCA reductive dechlorination. In other studies different bacteria within the same microbial community were shown to exhibit specific activities towards different chlorinated congeners [34]. Coherently with our results, Grostern and colleagues [35] described the co-growth of more microorganisms competing for the same chlorinated compound. As suggested by the authors, the co-growth probably occurs due to the presence of slightly different niches for each bacteria that avoid their direct competition. We hypothesize that the differences in niche partitioning in our culture are due to the presence of a suitable and not limiting growth substrate and electron donor. This enrichment conditions enhanced the growth of those portion of the microbial community that might function as a flexible reservoir of various degraders of halogenated compounds.

The third most abundant OTU was related to *Desulfitobacterium metallireducens*, identified for the first time by Finneran and colleagues [36] in an uranium-contaminated aquifer. *Desulfitobacterium metallireducens *was described as an anaerobic bacterium able to couple its growth to the reduction of metals, humic acids and chlorinated compounds (trichloroethylene or tetrachloroethylene) using lactate as electron donor [36]. However its involvement in 1,2-DCA dehalogenation was not demonstrated. The co-growth in dechlorinating enrichment cultures of bacteria not directly related to the dehalogenating activity but with an indirect role in the process is commonly reported in literature [37,38,26]. This is supported also by the identification of sequences related to *Lactobacillus *sp. and *Zymophilus *sp., species known to be able to produce lactate and other organic acids useful as electron donors for the reductive dechlorination process. The structure and composition of the microbial community suggested that the positive response to the treatment is possibly led by a 'complementarity effect' rather than a 'selection effect' [2].

A further sign of the 'complementarity effect' was the identification of possible functional redundancy related to 1,2-DCA reductive dechlorination. The RDs gene clone library showed four different RDs enzymes, all conserving signature residues possibly linked to 1,2-DCA dehalogenation [25,26]. Two main groups of RDs (cluster I and III) characterized the consortium and it was possible to connect the presence of these functional genes to the two most abundant OTUs identified in the 16S rRNA gene library. Sequences belonging to the group I were associated to *Desulfitobacterium dichloroeliminans *strain DCA1, that showed the same amino acid signatures in the two reductive dehalogenases in its genome [25]. Sequences of the group III most likely belonged to *Dehalobacter*. In fact, taking into account that i) *rdhA1 *of *Dehalobacter *sp. WL was found in a culture amended with only 1,2-DCA; ii) its abundance was shown to be correlated with both *Dehalobacter *growth and 1,2-DCA dechlorination activity; and that iii) *Dehalobacter *sp. WL *rdhA1 *transcription occurred upon exposure to 1,2-DCA, Grostern and colleagues [26] concluded that *rdhA1 *represents a putative *Dehalobacter *1,2-DCA RD gene. At the moment, regarding the RDs sequences of the groups II and IV, we have no definitive proof or literature data supporting their involvement in any specific dehalogenation process.

Finally, the occurrence of *Dehalobacter *sp. WL *rdhA2 *and *rdhA3 *in similar abundances in a 1,1,2-TCA-amended culture was correlated to a second *Dehalobacter *strain that uses 1,1,2-TCA and, to a lesser extent, 1,2-DCA [26]. Our findings could support this hypothesis, with strain WL *rdhA3 *that clusters together *rdhA1 *in group III (Figure 2) and *rdhA2 *completely separated from any other cluster and thus possibly not involved in 1,2-DCA degradation.

## Conclusions

The capacity of sharing metabolic functionalities between different members of a microbial community is a fundamental feature for the efficiency of a microbial community, especially under stress conditions. In fact, when a given species cannot stand the environmental fluctuations, the community functional stability is assured by the presence of other species capable of performing the same function [29,33]. This is of special interest for bioremediation treatments when normally the microbial communities have to face harsh environmental conditions with the potential toxic effect of the pollutant and the low availability of one or more carbon sources, electron donors or nutrients.

Here we showed the characterization of a 1,2-DCA degrading microbial consortium, providing the concept that microbial metabolism can be exploited to solve real practical problems (MRM).

Upon several sub-culturing steps providing 1,2-DCA and lactate as electron donor, we were able to enrich a microbial community that could degrade up to 900 ppm of 1,2-DCA in less then 15 days. This community was dominated by bacteria belonging to the Firmicutes phylum. Among them, the most abundant OTUs were related to *Desulfitobacterium dichloroeliminans *strain DCA1 and *Dehalobacter *sp. WL, two strains known to be able to reductively dechlorinate 1,2-DCA to ethene with no other toxic intermediate metabolite. The structure of the microbial community was also correlated to functional redundancy with the identification of four phylogenetically-distinct catalytic enzymes, all conserving signature residues possibly linked to 1,2-DCA dehalogenation.

In MRM terms, the application of lactate represents a possible solution to boost the potential of the autochthonous microbial community for the degradative activity. On the other side, the enriched consortium itself is a potential candidate inoculum for bioaugmentation purposes to support groundwater remediation when such metabolic capabilities are not naturally present [39]. Further studies are needed for the *in situ *validation of these principles.

## Methods

### Establishment of enrichment culture

Anaerobic microcosms were prepared with groundwater from an aquifer contaminated for more than 30 years by 1,2-DCA, as described by Marzorati and colleagues [12]. The groundwater was contaminated with 1,2-DCA at a high concentration (up to 900 mg L^-1^), but not by other chlorinated ethanes or ethenes. The bacterial dehalogenating consortium was maintained through adhesion of the original culture to an active-carbon support and the microcosms were prepared in an anaerobic box incubated under N_2_/CO_2_/H_2 _(80/15/5%) at room temperature (average temperature 22°C) and fed with 5 mM Na lactate and additional 1,2-DCA. The medium was prepared as previously reported by Marzorati and colleagues [12].

The concentrations of 1,2-DCA, ethene, vinyl chloride and other possible degradation products in the microcosms were analyzed by head-space gas chromatography, on a 7694 Agilent gas chromatograph equipped with a flame ionization detector set at 200°C on a DB624 column (J&W Scientific, Folsom, CA) at constant oven temperature at 80°C. 1,2-DCA limit of detection was 1 μg L^-1^.

### PCR amplification of 16S rRNA and reductive dehalogenase genes

DNA from the enrichment culture was extracted, starting from 1.5 mL of suspension, as previously described [40]. 16S rRNA gene were amplified using bacterial universal primers 27f (5'AGAGTTTGATCCTGGCTCAG 3') and 1494r (5'CTACGGCTACCTTGTTACGA 3') with the following reaction: 1× PCR buffer (Invitrogen, Milan, Italy), 1.5 mM MgCl_2_, 0.12 mM of each dNTP, 0.3 μM of each primer, 1 U of Taq polymerase in a final volume of 50 μL. Initial denaturation at 94°C for 5 min was followed by 5 cycles consisting of denaturation at 94°C for 1 min, annealing at 50°C for 1 min, and extension at 72°C for 2 min, and by 30 cycles consisting of denaturation at 94°C for 1 min, annealing at 55°C for 1 min, and extension at 72°C for 2 min. A final extension at 72°C for 10 min was added.

A region of the reductive dehalogenase gene cluster including all *dcaA *and 194 bp of *dcaB *gene was amplified using primers PceAFor1 (5' ACGTGCAATTATTATTAAGG3') and DcaB rev (5'TGGTATTCACGCTCCGA3'), as reported by Marzorati and colleagues [25].

### 16S rRNA and functional genes libraries construction

Sixty ng PCR product (insert:vector molar ratio of 3:1) was used for cloning reactions using the pGEM cloning kit (pGEM-T Easy Vector Systems, Promega, Milan, Italy) following the recommendations of the manufacturer. A direct PCR assay was performed on white colonies to amplify the insert using primers T7 (3'CTAATACGACTCACTATAGGG 5') and SP6 (3'ATTTAGGTGACACTATAGAATA 5'). Amplification reactions were performed in a 25 μL total volume containing, 1× reaction buffer (Invitrogen), 0.12 mM of each dNTP, 0.7 U of Taq DNA polymerase, 0.3 μM of each primer. Reactions were performed with the following protocol: an initial melting at 94°C for 4 min, followed by 30 cycles at 94°C for 30 s, 50°C for 1 min, 72°C for 1.5 min and a final extension step at 72°C for 5 min. PCR products were purified by a QIAquick PCR Purification Kit (Qiagen, Milan, Italy) according to the manufacturer's instructions. Clones from bacterial 16S rRNA gene library were sequenced with primer 27f, those from RD libraries with the primer PceAFor1, using the ABI Prism BigDye terminator cycle sequencing kit (Applied Biosystems, Milan, Italy) and an ABI 310 automated sequencer (Applied Biosystems). Rarefaction curves were built using the PAST program [41]. To determine the Operational Taxonomic Units (OTUs) the 99% identity criterion for the full-length 16S rRNA sequence has been chosen to minimize underestimation of the true functional diversity of the ecosystem [42] and to minimize PCR artifacts due to Taq DNA polymerase errors [43]. Two clones per each OTU were completely sequenced using primer 1494r for bacterial 16S rRNA gene libraries and DcaB rev and DHLF3 (5' GAAATAGCTAATGAGCCTGT 3') for RD gene libraries.

### Statistical analysis

The closest relative match to each sequence was obtained with BLASTN facilities [44] and Classifier v.6.0 program at the Ribosomal Database Project (RDP) 10 http://rdp.cme.msu.edu/classifier/classifier.jsp[45]. Chimera sequences were checked with the chimera-check tool at greengenes.lbl.gov [46]. Sequences were phylogenetically aligned using ClustalW software http://clustalw.genome.ad.jp/[47]. Alignments were checked manually and corrected for any misalignments that had been generated. Tree construction was done with Treecon v.1.3b using neighbor-joining methods and a bootstrap analysis with 1000 replicates. Matrix similarities among 16S rRNA sequences and RDs sequences were calculated in Phylip Interface using Jukes-Cantor algorithm for DNA and Dayhoff PAM matrix for protein sequences.

The following indices were calculated by using the PAST program [41] to give a more detailed description of the diversity within the samples [48]: Dominance (D = Σpi^2^), Shannon (H = -Σ(pi) × (log_e_pi) and Simpson (Ds = 1-D), where pi is the ratio between the abundance of each OTU and the total number of clones analyzed. In order to graphically represent the evenness of the bacterial communities, Pareto-Lorenz distribution curves [28] were set up based on the 16S rRNA gene clone library results as reported by Marzorati and colleagues [29].

### Sequence accession number

The nucleotide sequences of the clones identified in this study were deposited in the EMBL nucleotide sequence database (GenBank/EMBL/DDBJ) under the accession numbers from FM204994 to FM205005 for bacterial 16S rRNA genes and from FM204935 to FM204942 for RD sequences.

## Competing interests

The authors declare that they have no competing interests.

## Authors' contributions

DD, FdF and WV planned the work that led to the manuscript; MM, AB and LC produced and analyzed the experimental data; LW and GC participated in the interpretation of the results; MM, AB and DD wrote the paper; WV and FdF participated in the revision of manuscript's intellectual content. All authors read and approved the final manuscript.
